# Upregulated Polo-Like Kinase 1 Expression Correlates with Inferior Survival Outcomes in Rectal Cancer

**DOI:** 10.1371/journal.pone.0129313

**Published:** 2015-06-05

**Authors:** T. G. Tut, S. H. S. Lim, I. U. Dissanayake, J. Descallar, W. Chua, W. Ng, P. de Souza, J-S. Shin, C. S. Lee

**Affiliations:** 1 School of Medicine, University of Western Sydney, Liverpool, New South Wales 2170, Australia; 2 Ingham Institute for Applied Medical Research, Liverpool, New South Wales 2170, Australia; 3 University of New South Wales, Kensington, New South Wales 2052, Australia; 4 Department of Medical Oncology, Liverpool Hospital, Liverpool, New South Wales 2170, Australia; 5 Department of Anatomical Pathology, Liverpool Hospital, Liverpool, New South Wales 2170, Australia; 6 Bosch Institute, University of Sydney, Camperdown, New South Wales 2006, Australia; National Cancer Institute, NIH, UNITED STATES

## Abstract

**Background:**

Human polo-like kinase 1 (PLK1) expression has been associated with inferior outcomes in colorectal cancer. Our aims were to analyse PLK1 in rectal cancer, and its association with clinicopathological variables, overall survival as well as tumour regression to neoadjuvant treatment.

**Methods:**

PLK1 expression was quantified with immunohistochemistry in the centre and periphery (invasive front) of rectal cancers, as well as in the involved regional lymph nodes from 286 patients. Scores were based on staining intensity and percentage of positive cells, multiplied to give weighted scores from 1–12, dichotomised into low (0–5) or high (6–12).

**Results:**

PLK1 scores in the tumour periphery were significantly different to adjacent normal mucosa. Survival analysis revealed that low PLK1 score in the tumour periphery had a hazard ratio of death of 0.59 in multivariate analysis. Other predictors of survival included age, tumour depth, metastatic status, vascular and perineural invasion and adjuvant chemotherapy. There was no statistically significant correlation between PLK1 score and histological tumour regression in the neoadjuvant cohort.

**Conclusion:**

Low PLK1 score was an independent predictor of superior overall survival, adjusting for multiple clinicopathological variables including treatment.

## Introduction

Colorectal cancer is one of the leading causes of mortality in the developed world. Rectal cancers comprise a third of these cases and carry a worse prognosis than colon cancers. In the locally advanced setting, they are treated differently to colon cancers, with trimodality therapy consisting of neoadjuvant chemoradiation, surgery and adjuvant chemotherapy [1]. Rectosigmoid tumours are treated akin to colonic tumours, as is metastatic rectal cancer. There is a need for biomarkers to inform prognosis and choice of therapy, assess treatment response, and aid in the stratification of patient risk in order to adapt and personalise patient care.

Recently, Rodel and colleagues reported polo-like kinase 1 (PLK1) to be a novel predictive biomarker for radiation sensitivity in rectal cancer [2]. We hypothesise that over-expression of PLK1 correlates with poorer outcomes in rectal cancer. PLK1 is a mitotic serine/threonine kinase cell cycle regulator necessary for cell division, involved in the regulation of mitotic entry, spindle formation and cytokinesis [3–5]. The functional significance of PLK1 in carcinogenesis and malignant progression is not clearly understood, but nonetheless its overexpression is found in many cancer types [2,6], including colorectal cancer [7,8]. Its tumourigenic capability has been shown in nude mice injected with PLK1-overexpressing NIH3T3 fibroblasts [9]. Utilisation of small interfering RNA [10,11] and antisense oligonucleotides [12] in malignant cells to deplete PLK1 levels also induced apoptosis and containment of malignant proliferation in *in-vitro* and *in-vivo* models. PLK1 activity is necessary in repair from DNA damage resulting from chemo- and radiotherapy [13]. Hence PLK1 appears to be a promising predictive and prognostic biomarker, and herein we investigate its role in rectal cancer. We also aim to show that PLK1 is independent of the cell proliferation marker Ki67.

## Materials and Methods

Ethics approval was obtained on 22^nd^ June 2012 from the Sydney South-West Area Health Service Ethics Review Committee, reference number HREC/12/LPOOL/102. The institutional review board waived the need for written informed consent from the participants as the project was deemed to be in the low or negligible risk category. Information was de-identified prior to analysis.

Specimens from primary surgery for rectal or rectosigmoid cancers were obtained from the South-Western Area Pathology database, Australia from 2000–2010. Surgery consisted of total mesorectal excision, with anterior or abdominoperineal resection. Variables of interest included age, gender, pathological stage of tumour, grade, vascular invasion, perineural invasion, tumour-infiltrating lymphocytes and treatment. Staging was based on the American Joint Committee on Cancer (AJCC) tumour-node-metastases (TNM) system. Outcomes of interest were overall survival (OS) and histological tumour regression (TRG) in the resected bowel for cases treated with neoadjuvant chemoradiation. OS was defined as the time from diagnosis to last follow-up or death. TRG was graded based on the AJCC criteria, modified from Ryan [14]: complete response with no viable malignant cells (0), moderate response with single or small group of malignant cells (1), minimal response with residual malignancy outgrown by fibrosis (2) and poor response with extensive residual malignancy (3). RG 0, 1 and 2 were categorised as responders and TRG 3 as non-responders. Follow-up consisted of regular clinic visits, colonoscopy, blood tests and imaging at the discretion of the treating specialist.

For each patient, donor blocks of paraffin embedded tissue were retrieved from the anatomical pathology department. Two cores, each one millimetre in diameter, were obtained from each sampling site which included the tumour centre (TC), tumour periphery (TP), normal mucosa close to tumour (NCT) and normal mucosa away from tumour (NAT). Two tissue cores from lymph nodes (LN) in the node-positive cases were also obtained. TC was taken from centre of tumour mass, TP from the infiltrating/invasive tumour periphery, NCT from normal mucosa immediately adjacent to tumour and NAT from normal mucosa well away from tumour, usually at the end margins of the resected bowel. These were transferred into the pre-drilled wells in the tissue microarray (TMA) block using the Beecher Manual tissue arrayer-1 (Sun Prairie, WI, USA). The TMA blocks were heated for 5 minutes in 60°C oven to seal the gaps between tissue cores and surrounding paraffin.

Slide sections taken from the TMAs were deparaffinised with xylene, followed by absolute alcohol, then re-hydrated in alcohol gradient. Antigen was retrieved in heat in 98°C water bath with Tris EDTA (pH 9.0) buffer. Endogenous peroxidase activity was blocked with H_2_O_2_, prior to incubation with PLK1 primary monoclonal mouse antibody (1:50 dilution, sc17783, Santa Cruz Biotechnology). The antibody was sourced and stained as per previously published method [15]. Linker step with mouse linker solution, followed by secondary antibody incubation (Envision polymer FLEX/HRP, Dako) preceded the application of chromogen (Flex DAB, Dako) and counterstaining with haematoxylin. Ki67 was stained on the Dako Autostainer using the supplied antibody at 1:100 dilution, with heat induced epitope retrieval at pH 6.0 and 10 minute primary antibody incubation time.

**Fig 1:**
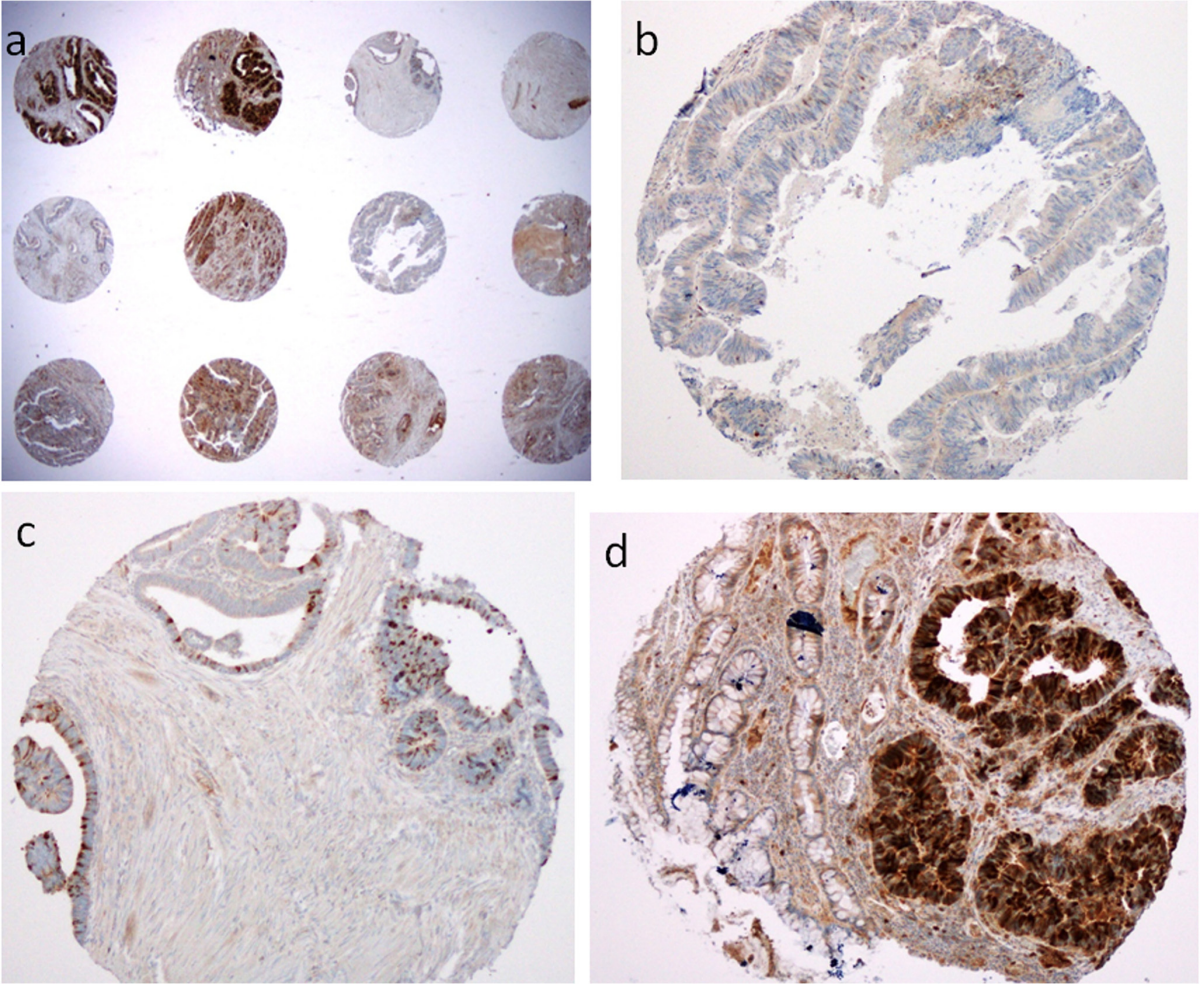
Polo-like kinase 1 immunohistochemical staining in rectal cancer cells in tissue microarrays in low power (a) and high power. High power images show scores of (b) weighted score of 0, (c) weighted score of 3, from 1 (percentage 5–25%) x 3 (strong intensity) and (d) weighted score of 4 (percentage >75%) x 3 (strong intensity), note the contrast with adjacent normal glands

The immunostained sections were examined by manual counting of cells in each TMA dot, with the observer blinded to clinical outcomes. Percentage of positive cells and staining intensity of PLK1 were scored (Fig 1). Intensity was graded as negative (0), weak (1+), moderate (2+) or strong (3+) and percentage of positive cells graded as <5% (0), 5–25% (1), 26–50% (2), 51–75% (3) and >75% (4). These two measures were multiplied to give weighted scores from 0–12, dichotomised into low (0–5) or high (6–12), as described by Rodel [15]. The two scores for each duplicate sampling site were averaged, obtaining the final average weighted scores (S1 Table). For Ki67, percentage staining was scored, and the two scores for each duplicate sampling site were averaged (S1 Table).

Paired t-test was used to compare PLK1 in TC and TP, Fisher’s exact test for associations between PLK1 and clinicopathologic variables, and Cox regression for OS. Only cases with complete data on all the variable domains were included in the univariate and multivariate analyses. Significance was set at *p*<0.05. Variables with a univariate *p*-value ≤0.2 were included in the initial multivariate model. Non-significant variables were removed iteratively and coefficients checked to determine whether removal of a variable resulted in a large effect change for any of the remaining variables. This was repeated until significant variables remained. Two way interactions between the remaining variables were checked for significance and included in the model if they were significant. The supremum test for proportional hazards assumption was used to determine whether the hazard functions over time were constant. For variables that did not satisfy the proportional hazards assumption, an interaction term between time and that variable was included into the model to account for non-proportionality. Subgroup analyses were performed using a similar procedure for patients in the lymph node positive group to determine if PLK1 in the involved node correlated with node number (Fisher’s exact test) and with survival (Cox regression). Another subgroup analysis (Fisher’s exact test) was performed for patients who had undergone neoadjuvant therapy to determine if there was a correlation between PLK1 and TRG. Ki67 was correlated with PLK1 scores using logistic regression. Data analysis was generated using SAS Enterprise Guide software, Version 6.1 of the SAS System for Windows (SAS Institute Inc., Cary, NC, USA).

## Results

### Characteristics of the study population

Two hundred and eighty-six cases were identified, with a median age of 73 years (S1 Table). The cohort consisted of 34% female, 66% male, 33% pT1/2, 67% pT3/4, 48% node-positive and 7% with metastatic disease. Median follow-up was 3.1 years and 5-year OS 58%. Twenty-two percent received neoadjuvant therapy and 30% adjuvant therapy. Adjuvant chemotherapy consisted of infusional 5-FU, capecitabine or FOLFOX (5-FU and oxaliplatin). Out of the neoadjuvant therapy group, 25% received short-course (25 Gy in 5 fractions, 5 Gy per fraction, over 5 days) and 75% long-course concurrent chemoradiation (45 to 50.4 Gy, 1.8 Gy per fraction, over 5 to 6 weeks, with concurrent infusional 5-fluorouracil 225mg/m^2^/day).

### PLK1 scores

**Fig 2:**
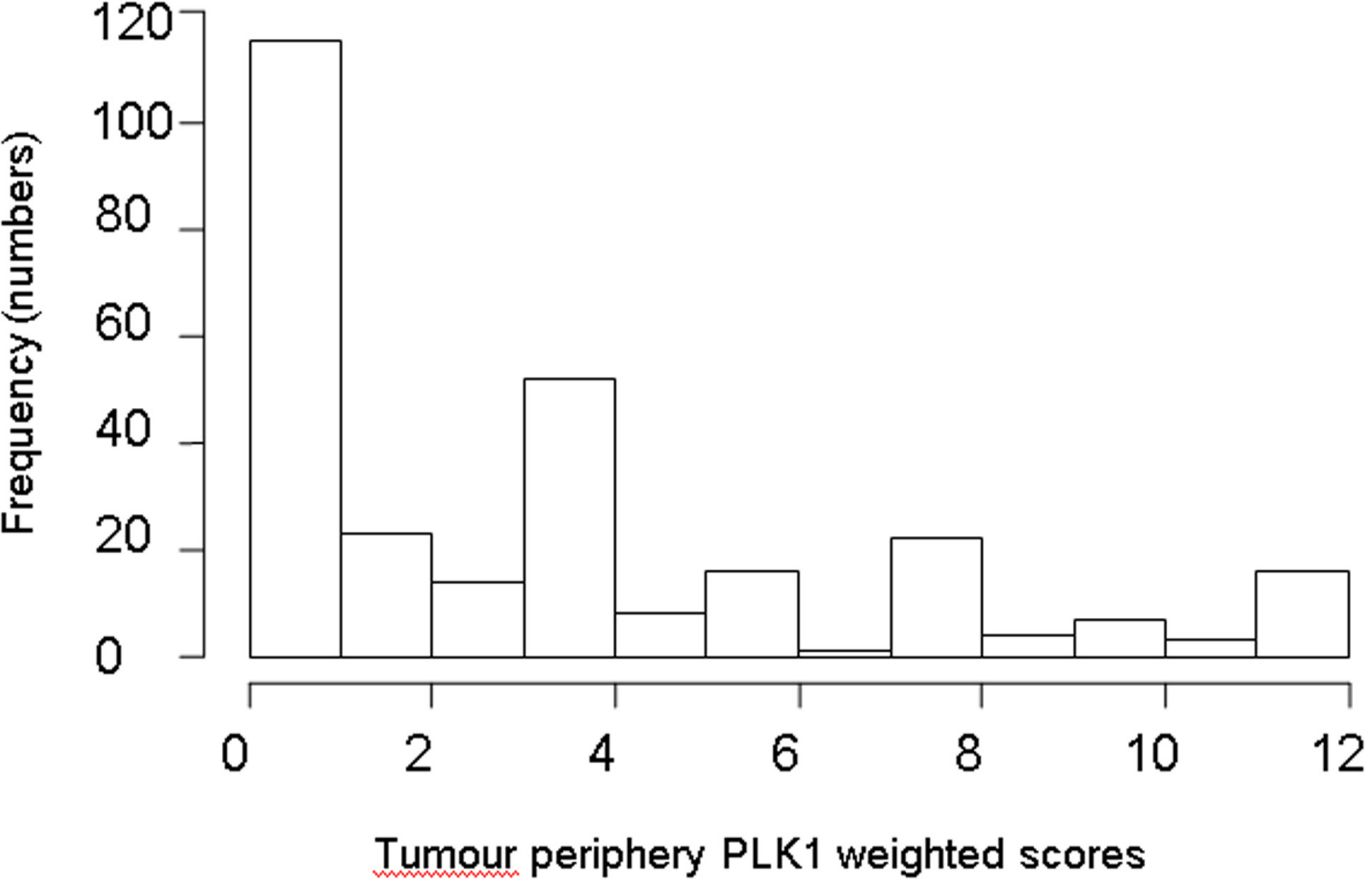
Histogram showing the distribution of the discrete tumour periphery (TP) PLK1 scores.

Mean weighted PLK1 score was 2.5 for tumour centre (TC) and 3.4 for tumour periphery (TP). The breakdown of TC and TP weighted scores is shown in Table 1 and the discrete scores shown in S1 Table. The dichotomised scores of positive versus negative are also shown. TC and TP weighted scores were significantly different (paired t-test *p*<0.001). Staining in TP and TC had similar proportions of >75% staining (i.e. 35%) however the proportions of 3+ staining was 14% in TC and 25% in TP. Mean PLK1 score was 3.4 for NCT and 3.0 for NAT, and these scores were significantly different (*p* = 0.025). NAT was significantly different to TP (*p* = 0.011) but not to TC (0.08), hence TP was deemed to be a more accurate representation of tumour staining, and will be used herein for analyses. The distribution of the TP scores is illustrated in Fig 2.

**Table 1:** Polo-like kinase 1 scores (immunohistochemistry) in rectal tumour centre and periphery.

Tumour centre				Tumour periphery			
Weighted score		Dichotomised score		Weighted score		Dichotomised score	
**0–5**	243 (85%)	**Negative**	117 (42%)	**0–5**	212 (74%)	**Negative**	91 (32%)
**6–12**	37 (13%)	**Positive**	163 (56%)	**6–12**	69 (24%)	**Positive**	190 (66%)
**Missing**	6 (2%)	**Missing**	6 (2%)	**Missing**	5 (2%)	**Missing**	5 (2%)
**Total**	286 (100%)	**Total**	286 (100%)	**Total**	286 (100%)	**Total**	286 (100%)

### Associations between PLK1 scores and clinicopathological characteristics

An association was found between TP scores and metastatic status, in that patients with metastatic disease had a low TP PLK1 score in 95% of the cases compared to 72% in the non-metastatic disease group (Table 2). There were no other significant associations.

**Table 2:** Fisher’s test of association between tumour periphery (TP) polo-like kinase 1 (PLK1) scores and clinicopathological variables.

Variable	Number	Low TP PLK1 score (0–5)	High TP PLK1 score (6–12)	*p*-value
***Gender***				0.363
Male	186	76%	24%	
Female	95	74%	26%	
***Age***				0.527
Less than median	139	76%	25%	
More than median	141	75%	25%	
***Tumour stage***				0.478
T1 and T2	91	75%	25%	
T3 and T4	190	76%	24%	
***Nodal stage***				0.461
Negative	146	76%	24%	
Positive	135	75%	25%	
***Metastatic status***				0.021
Absent	234	72%	28%	
Present	19	95%	5%	
***Neoadjuvant therapy***				0.242
No	206	73%	27%	
Yes	53	79%	21%	
***Grade***				0.378
1 and 2	260	75%	25%	
3	21	81%	19%	
***Vascular invasion***				0.520
Absent	213	76%	24%	
Present	68	75%	25%	
***Perineural invasion***				0.534
Absent	233	76%	25%	
Present	48	75%	25%	
***Adjuvant treatment***				0.318
No	163	76%	24%	
Yes	72	72%	28%	
**Tumour-infiltrating lymphocytes**				0.256
Absent	257	77%	23%	
Present	22	68%	32%	

### Survival analyses

**Fig 3:**
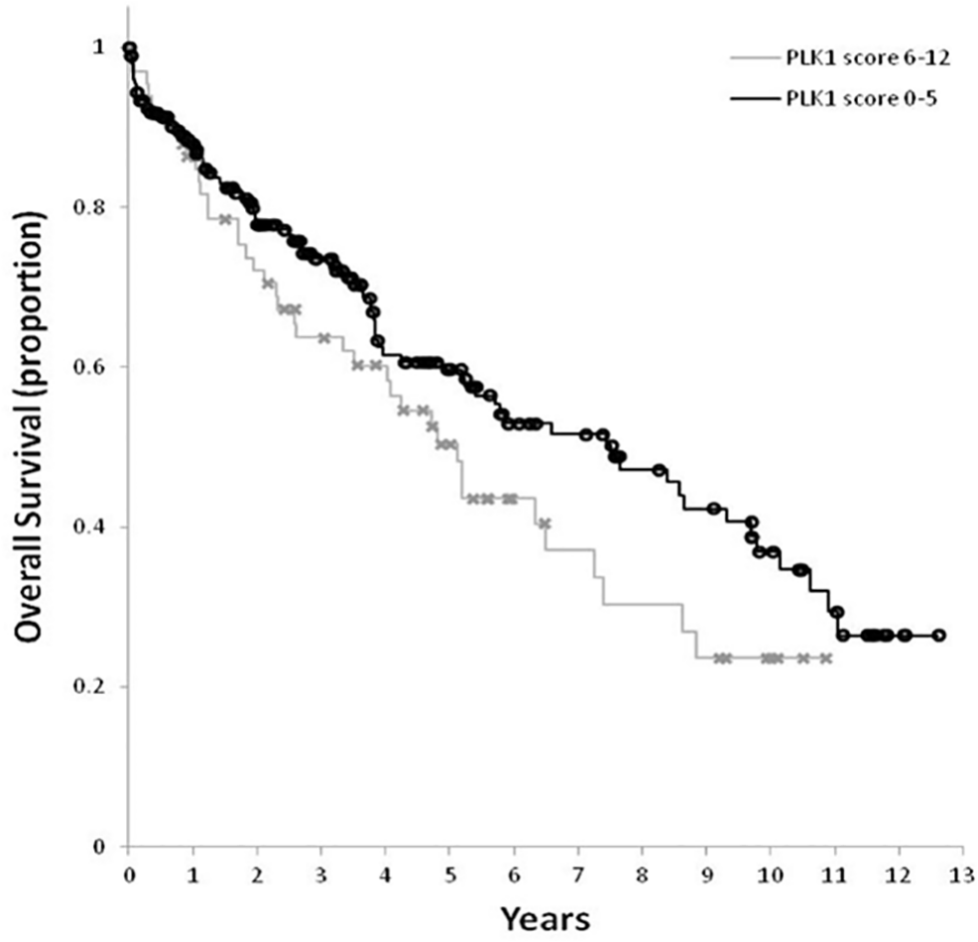
Kaplan Meier survival curve for tumour peripheral (TP) polo-like kinase 1 (PLK1) weighted scores.

The 12 variables of interest included TP PLK1 weighted score, age, gender, TNM stage, histological grade, vascular invasion, perineural invasion, presence of tumour-infiltrating lymphocytes, adjuvant chemotherapy and neoadjuvant treatment. There were 219 cases with complete data on all these domains. Cox regression univariate analyses showed higher TP PLK1 score, higher age, higher tumour T stage, metastatic status, presence of vascular and perineural invasion and absence of adjuvant chemotherapy to be significant predictors of worse OS (Table 3, Fig 3). The final multivariate model includes TP PLK1 score, nodal status, metastatic status, an interaction between adjuvant chemotherapy and vascular status, and an interaction between adjuvant chemotherapy and time (Table 3). High TP PLK1 score, positive nodal status, and presence of metastasis were found to be associated with worse survival. Patients who had not received adjuvant chemotherapy had worse survival than patients who had received adjuvant chemotherapy and this effect was observed to be worse for patients whose cancers had vascular invasion. Over time, the effect of not having adjuvant chemotherapy reduced, and was non-significant after 2 years for cancers without vascular invasion. However, survival remained significantly worse for up to 10 years in cancers with vascular invasion (S1 Fig).

**Table 3:** Univariate analyses and multivariate model of tumour periphery (TP) polo-like kinase 1 (PLK1) scores with clinicopathological variables for survival outcomes.

	Univariate			Multivariate			
Variable	Hazard Ratio	CI	*p*-value	Coefficient	Hazard Ratio	CI	*p*-value
**TP PLK1**							
Less than 6	0.72	(0.46, 1.12)	0.1452	-0.52	0.59	(0.38, 0.94)	0.0245
6 or more	Reference						
**Gender**							
Male	0.96	(0.62, 1.49)	0.8649				
Female	Reference						
**Age**							
Less than median	0.57	(0.37, 0.88)	0.0121				
More than median	Reference						
**Tumour Stage**							
T1 and T2	0.59	(0.38, 0.94)	0.0249				
T3 and T4	Reference						
**Nodal Status**							
Negative	0.69	(0.45, 1.04)	0.0773	-0.57	0.57	(0.36, 0.9)	0.0167
Positive	Reference						
**Metastatic Status**							
Absent	0.16	(0.07, 0.33)	<0.0001	-1.22	0.29	(0.12, 0.7)	0.0054
Present	Reference						
**Neoadjuvant treatment**							
No	1.07	(0.62, 1.88)	0.8034				
Yes	Reference						
**Adjuvant Chemotherapy**							
No	2.05	(1.22, 3.43)	0.0068	3.69			<0.0001
Yes	Reference						
**Grade**							
1 and 2	0.54	(0.26, 1.11)	0.0939				
3	Reference						
**Vascular Invasion**							
Absent	0.49	(0.31, 0.76)	0.0015	0.82			0.2479
Present	Reference						
**Perineural Invasion**							
Absent	0.46	(0.28, 0.75)	0.0016				
Present	Reference						
**TIL**							
Absent	2.33	(0.94, 5.75)	0.0674				
Present	Reference						
**Adjuvant chemotherapy** * **Vascular Invasion**							
No Adjuvant chemotherapy, No Vascular invasion				-2.36			0.0001
**Adjuvant Chemotherapy** * **Time**				-0.21			0.023

Abbreviations: CI = confidence intervals, PLK1 = polo-like kinase 1, TIL = tumour-infiltrating lymphocytes, TP = tumour periphery

* denotes interaction

### Lymph node positive subgroup

Mean LN PLK1 score in the 105 node-positive cases was 3.1. There was no statistically significant association between LN PLK1 scores and number of nodes involved (*p* = 0.097). However there appeared to be a trend between higher LN PLK1 scores and greater number of positive nodes, with 68% of high PLK1 scores having N2 compared to N1 status. There were borderline associations between LN PLK1 scores and vascular invasion (*p* = 0.064), perineural invasion (*p* = 0.070) and histological grade (*p* = 0.057).

In univariate analyses (n = 78), LN PLK1 was not significantly associated with survival (*p* = 0.114). In multivariate analyses (n = 78), LN PLK1 was not significantly associated with survival (*p* = 0.7083) after adjusting for adjuvant chemotherapy and vascular invasion interaction; as well as adjuvant chemotherapy and time interaction.

### Neoadjuvant subgroup

Out of the 57 patients who underwent neoadjuvant chemoradiation. Twenty-three percent had TRG grades 1 to 2 (responders) and 77% had TRG3 (non-responders). There was no significant association between TP PLK1 score and TRG (*p* = 0.096).

### Association between PLK1 and Ki67

Ki67 mean scores in TC, TP and LN were 9%, 8% and 4% respectively. cores in NAT and NCT were <1%. Logistic regression revealed no significant associations between PLK1 and Ki67 in TC (*p* = 0.539), TP (*p* = 0.406) and LN (*p* = 0.413).

## Discussion

While there are a number of publications regarding prognostic effects of PLK1 in various human malignancies [6,16], this is the first report to our knowledge, investigating PLK1 expression in different sampling sites from the same tumour, and demonstrating that upregulated PLK1 expression in the periphery of the primary tumour correlates with inferior survival outcome in rectal cancer, and is independent of proliferative marker Ki67.

We found variable expression of PLK1 in the centre and periphery of the primary tumour, and in nodal deposits. Compared to normal mucosa away from the tumour, the peripheral PLK1 score was significantly higher. The peripheral invasive front would be rationalised as being more reflective of tumour behaviour as it is more biologically active with regard to interaction with and destruction of the surrounding tissues. The periphery of tumour mass is better oxygenated and expected to be more responsive to radiation as opposed to the centre which may be ischaemic or necrotic. We propose that the tumour periphery be used as the sampling site for rectal tumour PLK1 scoring.

We demonstrated that TP weighted PLK1 score correlated with overall survival, with a HR of 0.59. The 5-year OS was 60% in the group with low PLK1 scores compared to 50% in the group with high PLK1 scores. Significant variables for survival included age, tumour stage, metastatic status and interactions between adjuvant chemotherapy with vascular invasion, and with adjuvant chemotherapy and time. TP PLK1 score was an independent prognostic factor of survival, even after adjusting for these variables. Importantly, we have adjusted for adjuvant treatment. Our median follow-up was 3.1 years, which would be a reflection of longer term survival given that three-year follow up is an appropriate endpoint with a high correlation with overall survival in colorectal cancer [17].

PLK1 expression has also been documented in precursor/early cancers of other organs, such as in ovarian cystadenoma [18] and in early papillary thyroid carcinoma [19] and previously PLK1 over-expressing colorectal cancers have been reported to display a more radioresistant phenotype [2]. We could not confirm this in our findings, however we recognise that the neoadjuvant cohort comprised only a small subset of our study population and hence may have insufficient power to demonstrate this relationship.

We also investigated PLK1 in lymph nodes in the node-positive population. There appeared to be a trend toward an association between higher LN PLK1 score and higher node number, which is consistent with our hypothesis that over-expression of PLK1 correlates with poorer outcomes. However LN PLK1 did not correlate significantly with survival, within the limitations of small subgroup analyses.

Low PLK1 score was significantly associated with presence of metastatic disease. Given that our cohort consisted of a small percentage of metastatic cases (7%), the clinical utility of PLK1 will need to be explored further in this patient population. It may be hypothesized that the majority of metastatic cases did not allow for the collection of resected tissue, hence limiting the ability to detect cases with high PLK1 staining. Larger studies are needed to define the interaction between PLK1 score and metastatic status with regard to outcomes.

We also performed corroborative staining with Ki67, and demonstrated no significant association between PLK1 and Ki67 staining. Hence PLK1, though important in cell cycle regulation, is not simply a surrogate for cell proliferation.

Our findings are consistent with observations from cell line studies, where re-instatement of PLK1 activity is essential in recovery from G2/M checkpoint arrest following DNA damage. The latter inhibits PLK1 which normally requires phosphorylation in its activation loop threonine 210 by Bora in collation with upstream kinase. Threonine 210 phosphorylation of PLK1 peaks with the normal accumulation of Bora in G2 phase. PLK1 subsequently activates downstream targets and cells progress through mitosis [20]. Continued PLK1 inactivation results in delayed entry to mitosis. By contrast, overexpression of constitutively phosphorylated PLK1 overrules the DNA damage induced G2/M checkpoint arrest [2,21,22]. This is important in rectal cancer as radiation induces double-stranded DNA breaks. Chemotherapy agents used in rectal cancer, either concurrent with radiation or in the adjuvant setting, include 5-fluoropyrimidine which is an anti-metabolite, and oxaliplatin which is an alkylating agent that causes cross-links in DNA.

Current therapeutic management of rectal cancer can be improved by the availability of better predictive and prognostic biomarkers. Carcinoembryonic antigen (CEA) is currently the only biomarker in the National Comprehensive Cancer Network (NCCN) guidelines, as part of baseline staging and in post treatment surveillance. In the metastatic setting, *K-ras* is a predictive marker for response to EGFR inhibitor therapy [23]. Our results suggest that PLK1 is a potentially useful prognostic biomarker in rectal cancer patients. PLK1 inhibition is currently considered a promising anticancer therapeutic agent. The PLK1 inhibitor Volasertib (BI6727) has completed phase II clinical trial to determine its anti-malignant and safety profiles [24,25]. Other PLK1 inhibitors in development include BI2536 (Axon), which if delivered before or after radiation exposure, could respectively result in either increased cell death during mitotic arrest or increased cell recovery following DNA repair during G2 checkpoint arrest [13]. Further prospective studies validating the clinical utility of PLK1, as demonstrated here, in rectal cancer management is needed to reinforce our findings and translate them into clinical use.

## Supporting Information

S1 Fig Effect of adjuvant chemotherapy stratified by vascular invasion status over time, HR = hazard ratio. (TIF)

S1 Table The dataset showing the 286 patients, including their discrete average PLK1 scores, Ki67 values, clinicopathological variables and survival outcomes. (XLS)

## References

[1] Sauer R, Liersch T, Merkel S, Fietkau R, Hohenberger W, Hess C, et al (2012) Preoperative versus postoperative chemoradiotherapy for locally advanced rectal cancer: results of the German CAO/ARO/AIO-94 randomized phase III trial after a median follow-up of 11 years. J Clin Oncol 30: 1926–1933. 10.1200/JCO.2011.40.1836 22529255

[2] Rodel F, Keppner S, Capalbo G, Bashary R, Kaufmann M, Rodel C, et al (2010) Polo-Like Kinase 1 as Predictive Marker and Therapeutic Target for Radiotherapy in Rectal Cancer. American Journal of Pathology 177: 918–929. 10.2353/ajpath.2010.100040 20581060 PMC2913372

[3] Reinhardt HC, Yaffe MB (2013) Phospho-Ser/Thr-binding domains: navigating the cell cycle and DNA damage response. Nature Reviews Molecular Cell Biology 14: 563–580. 10.1038/nrm3640 23969844

[4] van Vugt MATM, Bras A, Medema RH (2004) Polo-like kinase-1 controls recovery from a G2 DNA damage-induced arrest in mammalian cells. Molecular Cell 15: 799–811. 15350223 10.1016/j.molcel.2004.07.015

[5] van Vugt MATM, Medema RH (2005) Getting in and out of mitosis with Polo-like kinase-1. Oncogene 24: 2844–2859. 15838519 10.1038/sj.onc.1208617

[6] Takai N, Hamanaka R, Yoshimatsu J, Miyakawa I (2005) Polo-like kinases (Plks) and cancer. Oncogene 24: 287–291. 15640844 10.1038/sj.onc.1208272

[7] Han DP, Zhu QL, Cui JT, Wang PX, Qu S, Cao QF, et al (2012) Polo-like kinase 1 is overexpressed in colorectal cancer and participates in the migration and invasion of colorectal cancer cells. Medical Science Monitor 18: Br237–Br246. 22648245 10.12659/MSM.882900 PMC3560731

[8] Weichert W, Kristiansen G, Schmidt M, Gekeler V, Noske A, Niesporek S, et al (2005) Polo-like kinase 1 expression is a prognostic factor in human colon cancer. World Journal of Gastroenterology 11: 5644–5650. 16237758 10.3748/wjg.v11.i36.5644 PMC4481481

[9] Smith MR, Wilson ML, Hamanaka R, Chase D, Kung HF, Longo DL, et al (1997) Malignant transformation of mammalian cells initiated by constitutive expression of the polo-like kinase. Biochemical and Biophysical Research Communications 234: 397–405. 9177283 10.1006/bbrc.1997.6633

[10] Liu XQ, Erikson RL (2003) Polo-like kinase (Plk)1 depletion induces apoptosis in cancer cells. Proceedings of the National Academy of Sciences of the United States of America 100: 5789–5794. 12732729 10.1073/pnas.1031523100 PMC156279

[11] Spankuch-Schmitt B, Bereiter-Hahn A, Kaufmann M, Strebhardt K (2002) Effect of RNA silencing of polo-like kinase-1 (PLK1) on apoptosis and spindle formation in human cancer cells. Journal of the National Cancer Institute 94: 1863–1877. 12488480 10.1093/jnci/94.24.1863

[12] Spaekuch B, Steinhauser I, Wartlick H, Kurunci-Csacsko E, Strebhardt KM, Langer K (2008) Downregulation of plk1 expression by receptor-mediated uptake of antisense oligonucleotide loaded nanoparticles. Neoplasia 10: 223–234. 18320067 10.1593/neo.07916 PMC2259452

[13] Lund-Andersen C, Patzke S, Nähse-Kumpf V, Syljuåsen R (2014) PLK1-inhibition can cause radiosensitization or radioresistance dependent on the treatment schedule. Radiother Oncology (13). 10.1016/j.radonc.2013.12.014 24502970

[14] Ryan R, Gibbons D, Hyland JM, Treanor D, White A, Mulcahy HE, et al (2005) Pathological response following long-course neoadjuvant chemoradiotherapy for locally advanced rectal cancer. Histopathology 47: 141–146. 16045774 10.1111/j.1365-2559.2005.02176.x

[15] Rodel F, Keppner S, Capalbo G, Bashary R, Kaufmann M, Rodel C, et al (2010) Polo-like kinase 1 as predictive marker and therapeutic target for radiotherapy in rectal cancer. Am J Pathol 177: 918–929. 10.2353/ajpath.2010.100040 20581060 PMC2913372

[16] Takahashi T, Sano B, Nagata T, Kato H, Sugiyama Y, Kunieda K, et al (2003) Polo-like kinase 1 (PLK1) is overexpressed in primary colorectal cancers. Cancer Science 94: 148–152. 12708489 10.1111/j.1349-7006.2003.tb01411.x PMC11160284

[17] Sargent DJ, Wieand HS, Haller DG, Gray R, Benedetti JK, Buyse M, et al (2005) Disease-free survival versus overall survival as a primary end point for adjuvant colon cancer studies: individual patient data from 20,898 patients on 18 randomized trials. J Clin Oncol 23: 8664–8670. 16260700 10.1200/JCO.2005.01.6071

[18] Weichert W, Denkert C, Schmidt M, Gekeler V, Wolf G, Kobel M, et al (2004) Polo-like kinase isoform expression is a prognostic factor in ovarian carcinoma. British Journal of Cancer 90: 815–821. 14970859 10.1038/sj.bjc.6601610 PMC2410182

[19] Ito Y, Miyoshi E, Sasaki N, Kakudo K, Yoshida H, Tomoda C, et al (2004) Polo-like kinase 1 overexpression is an early event in the progression of papillary carcinoma. British Journal of Cancer 90: 414–418. 14735186 10.1038/sj.bjc.6601540 PMC2409566

[20] Qin B GB, Yu J, Yuan J, Lou Z. (2013) Ataxia telangiectasia-mutated- and Rad3-related protein regulates the DNA damage-induced G2/M checkpoint through the Aurora A cofactor Bora protein. J Biol Chem 288: 16139–16144. 10.1074/jbc.M113.456780 23592782 PMC3668769

[21] Smits VAJ, Klompmaker R, Arnaud L, Rijksen G, Nigg EA, Medema RH (2000) Polo-like kinase-1 is a target of the DNA damage checkpoint. Nature Cell Biology 2: 672–676. 10980711 10.1038/35023629

[22] van Vugt MATM Smits VAJ, Klompmaker R Medema RH (2001) Inhibition of polo-like kinase-1 by DNA damage occurs in an ATM- or ATR-dependent fashion. Journal of Biological Chemistry 276: 41656–41660. 11514540 10.1074/jbc.M101831200

[23] Karapetis CS, Khambata-Ford S, Jonker DJ, O'Callaghan CJ, Tu D, Tebbutt NC, et al (2008) K-ras mutations and benefit from cetuximab in advanced colorectal cancer. N Engl J Med 359: 1757–1765. 10.1056/NEJMoa0804385 18946061

[24] Francescangeli F, Patrizii M, Signore M, Federici G, Di Franco S, Pagliuca A, et al (2012) Proliferation state and polo-like kinase1 dependence of tumorigenic colon cancer cells. Stem Cells 30: 1819–1830. 10.1002/stem.1163 22753241

[25] Strebhardt K (2010) Multifaceted polo-like kinases: drug targets and antitargets for cancer therapy. Nat Rev Drug Discov 9: 643–660. 10.1038/nrd3184 20671765

