# Supplementary figures and images for: Upregulated Polo-Like Kinase 1 Expression Correlates with Inferior Survival Outcomes in Rectal Cancer
Source: PLoS One. 2015 Jun 5;10(6):e0129313. doi: 10.1371/journal.pone.0129313 (PMC4457812; doi:10.1371/journal.pone.0129313)

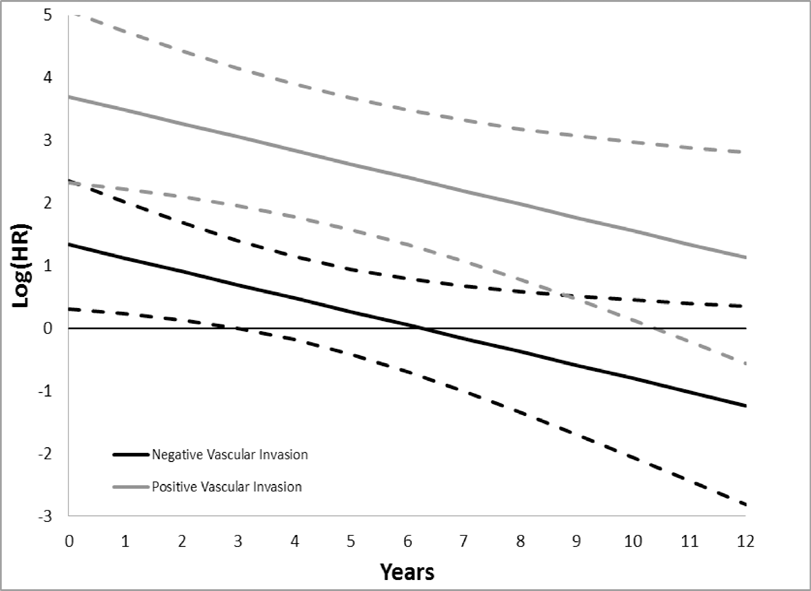

Supplement: S1 Fig — (TIF) [file pone.0129313.s001.tif]
